# Performance and Interfacial Microstructure of Al/Steel Joints Welded by Resistance Element Welding

**DOI:** 10.3390/ma17040903

**Published:** 2024-02-15

**Authors:** Nannan Wang, Jinpeng Li, Wenjie Wu, Xiaohui Bao, Kexu Ren, Jianghui Zhao, Huai Yao, Ranfeng Qiu

**Affiliations:** 1 Guanghan Campus, Civil Aviation Flight University of China, Guanghan 618307, China; 15896555218@163.com (N.W.); cafuclyljp@126.com (J.L.); wuwenjie@cafuc.edu.cn (W.W.); baoxiaohui@126.com (X.B.); 18437953616@163.com (K.R.); 2 Luoyang College, Civil Aviation Flight University of China, Luoyang 471000, China; 3 Key Laboratory of Flight and Operation of General Aviation Training, Luoyang 471000, China; 4 School of Materials Science and Engineering, Henan University of Science and Technology, Luoyang 471023, China; 15937128390@163.com (J.Z.); yaohuaitougao@126.com (H.Y.)

**Keywords:** aluminum, mild steel, resistance element welding, temperature history, interfacial reaction layer

## Abstract

In this study, an upper sheet of an A6061 aluminum alloy and a lower sheet of Q235 steel were welded by resistance element welding with a steel rivet. The temperature field during welding was calculated using ABAQUS numerical simulation software, and the interfacial microstructure was observed. A nugget was formed between the rivet shank and the lower sheet. With increases in welding current and welding time, the tensile shear load of the joint increased first and then decreased slightly. When the welding current was 14 kA and the welding time was 300 ms, the tensile shear load of the joint reached a maximum of 7.93 kN. The smaller the distance from the position to the lower sheet along the interface between the rivet shank and upper sheet, the longer the high-temperature duration and the higher the peak temperature during welding. At the junction of the rivet shank, upper sheet, and lower sheet in the joint, the high-temperature duration was the longest, at about 392 ms, and the peak temperature was the highest, at about 1237 °C. The results show that the smaller the distance from the position to the lower sheet along the interface between the rivet shank and the upper sheet in the joint, the thicker the reaction layer generated there, and that the thickness of the reaction layer was about 2.0 μm at the junction of the rivet shank, upper sheet, and lower sheet in the joint.

## 1. Introduction

For the purpose of lightweight automobiles, more and more lightweight materials, such as Al alloys, will be used in automobile bodies [1]. In this context, the joining between the lightweight metal Al alloy and the basic material steel is indispensable in automobile bodies [2,3]. In view of the fact that an automobile body is a typically welded thin-plate structural part, it is necessary to weld Al alloy and steel using resistance spot welding (RSW) that is suitable for the welding of thin plates [4,5]. However, the performance of resistance spot-welded (RSWed) Al/steel joints is poor, which is due to the formation of a thicker intermetallic compound (IMC) layer at the welding interface because of the local melting of base material during welding [6]. In order to suppress the growth of IMCs at the interface of RSWed Al/steel joints, some methods, such as controlling interface metallurgical reactions by use of an interlayer and optimizing the welding temperature field by changing the electrode cap shape, have been used in the RSW of Al/steel and extensively studied [7,8,9,10,11,12]. The results show that the adverse effect of IMCs on the performance of RSWed Al/steel joints has not been eradicated.

Recently, the joining of Al/steel with a hybrid method of riveting and RSW has been widely considered to further improve the performance of RSWed Al/steel joints. To overcome the technical limitations of RSW in the joining of dissimilar metals, Zhang et al. used a clinching method to punch a cylindrical rivet into a workpiece and pointed electrodes at the rivet and then performed RSW to weld DQSK steel and an AA5754 Al alloy [13]. Heidrich, et al. used self-piercing riveting to drive a semi-hollow rivet into an Al alloy sheet from one side, and then, the rivet leg formed a flange on its other side, thus obtaining an Al–rivet assembly [14]. After that, the Al–rivet assembly and a steel sheet were welded by using RSW, while the rivet leg flange acted as an interlayer; thus, a solid joint of Al/steel was obtained [14]. Lou et al. used the RSW method to heat a self-piercing riveted AA6061/DP590 joint to strengthen it [15]. For the same purpose, a method called resistance element welding (REW) was proposed for welding Al alloys and steel [16,17]. REW is carried out in two steps: First, the element is pressed into a prefabricated hole in the upper sheet. Second, the upper sheet, lower sheet, and rivet are welded together using RSW. To explore the influence of element shape on joint performance, rivets of various shapes were selected as elements for welding an Al alloy and steel [18,19]. The REW of dissimilar materials can realize a one-step integrated process by optimizing the geometry of the rivet’s shank tip [20]. Therefore, REW is a kind of method with great potential for the joining of dissimilar metals, and it is necessary to study this technology systematically.

Previous studies have shown that the essence of strengthening REW joints of dissimilar materials requires that a nugget is formed between the lower sheet and a rivet of homogeneous metal [21]. However, an IMC layer can also be found at the interface between the rivet shank and the upper sheet. In the present study, REW between an A6061 Al alloy and Q235 mild steel is carried out with a steel rivet, and the distribution characteristics of the IMCs layer at the interface between the rivet shank and the upper sheet combined with calculated temperature history curves are analyzed. The objective of this study is to find out the relationship between the IMC layer thickness at several feature regions and the thermal history within the joint to provide technical support for engineering applications of REW joints of Al/steel.

## 2. Experimental Materials and Procedures

In the present study, the experimental plan is to first select the materials to be welded and the rivet as an element, establish the welding process parameters, and then implement welding. After that, tensile shear testing and microstructure observation are carried out for the joints. On the other hand, the thermal history curves at feature regions in the joints are calculated using an established model [22].

The experimental materials used in the study were Q235 mild steel and an A6061 Al alloy; they were used as the lower sheet and upper sheet, respectively. The thickness of plates used for automobile bodies is generally 0.8~2.5 mm. As an exploratory study, plates with a thickness of 2 mm were selected for welding in this study. According to the relevant standard [23], they were cut to 100 mm × 30 mm. Q235 steel rivets of the same material as the lower sheet were selected as elements. Since the diameter of the electrode tip used was 6 mm, rivets with a shank diameter of 8 mm were selected for welding. According to the relevant standard [24], the diameter and thickness of the rivet tip are 12 mm and 2 mm, respectively. The rivet shank length is the same as the thickness of the upper sheet, which is 2 mm. 

The nominal compositions are Fe-1.0Mn-0.4Si-0.14C-0.04P-0.02S-0.06V for Q235 andAl-1.0Mg-0.6Si-0.3Cu-0.15Mn-0.15Ti-0.04Cr (wt-%) for A6061.

**Figure 1:**
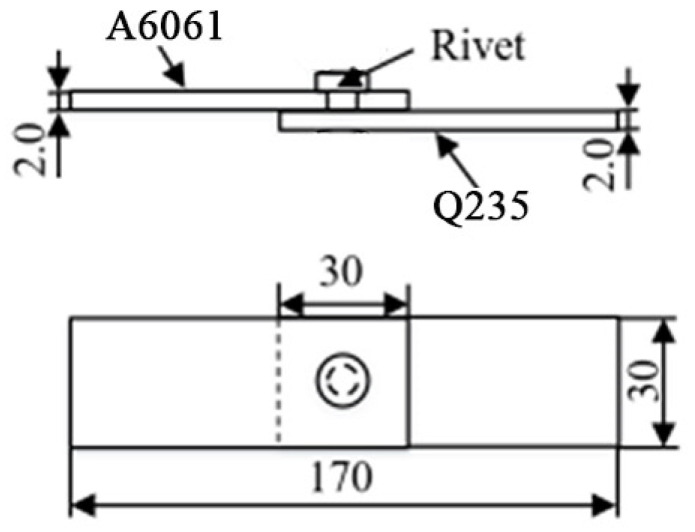
Assembly diagram of sample for tensile shear testing.

Before welding, a hole with a diameter of 8 mm (the same as the rivet shank diameter) was machined using a vertical lifting table milling machine (XA5032, BYJC, Beijing, China) in the center of the welding zone of the A6061 plate. The A6061 plates, Q235 steel plates, and rivets to be welded were cleaned with acetone and then air-dried and assembled into specimens, as shown in Figure 1, according to the standard [23]. The welding was implemented by use of a medium-frequency inverter RSW machine (DM200, Medar, Shanghai, China). The lower sheet was placed on the side of the lower electrode, and then, the upper electrode and lower electrode were aligned with the rivet in the axis direction. For resistance welding, welding current (WC) and welding time (WT) are the two most important parameters. Therefore, the WC and WT were accordingly changed for welding. Other parameters were selected according to experience when changing WC. The WC was determined according to the tensile shear load of the joints welded with various WTs when the WT was changed. The specific welding parameters used in the experiment are listed in Table 1. 

For each combination of parameters, seven joints were welded: five for tensile shear testing and two for microstructure observation. Under a cross-head velocity of 1.7 × 10^−5^ m/s, the tensile shear testing was carried out by using a universal testing machine (AG-1205 kN, Shimadzu, Kyoto, Japan). The microscopic observation samples were made by first cutting the joints along the rivet tip diameter with a numerical control wire cutting machine (DK7732, Jiangsu Dongqing, Taizhou, China), and then grinding and polishing their cross-section. Scanning electron microscopy (SEM, JSM-6300, JEOL, Tokyo, Japan) combined with energy dispersive X-ray spectroscopy (EDX, EDAX, Phoenix, AZ, USA) was used to observe the interfacial regions in the joint. Observation using an optical microscope (AxioVert A1, ZEISS, Oberkochen, Germany) at the nugget zone was performed after the cross-section was etched using a 4% nitrate alcohol solution.

ABAQUS 6.14.44 software was used to simulate the calculation of the temperature field during REW between A6061 and Q235 steel. The modeling details have been reported in reference [22].

## 3. Experimental Results and Discussion

**Figure 2:**
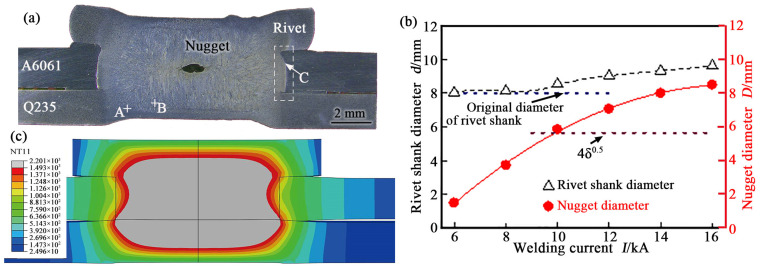
Cross-sectional photograph of REW A6061/Q235 joint (**a**); influence of WC on the rivet shank diameter and nugget diameter of REW A6061/Q235 joint (**b**); simulated cross-section (**c**).

Figure 2a shows a cross-sectional photograph of the joint between the A6061 Al alloy and Q235 mild steel welded by REW (hereafter called the REW A6061/Q235 joint), which was manufactured at a WC of 14 kA, a WT of 300 ms, and an electrode pressure of 3 kN. Four features can be observed in Figure 2a. Firstly, a nugget was observed in the joint, which made the rivet shank and the lower Q235 sheet join tightly together. During the welding, the resistance heat generated caused the partial metal of the rivet and the lower sheet to melt and form the nugget after solidification. In the direction of plate thickness, most of the nugget was located inside the rivet shank. This is a result of the influence of the water-cooled electrodes. In the welding process, the water-cooled electrodes were used for heat dissipation to ensure that the electrodes did not adhere to the workpieces. This resulted in the local metal adjacent to the electrodes not melting during welding. Therefore, the growth of the nugget in the direction of plate thickness was mainly towards the rivet. Meanwhile, the nugget also horizontally grew, which formed a nugget with a certain diameter. The nugget size is related to the WC used. Figure 2b shows the influence of the WC on the nugget diameter of the REW A6061/Q235 joint. The nugget diameter increased with an increase in WC. When the WC was greater than 10 kA, the nugget diameter was larger than 4δ^0.5^ (δ is the thickness of the plate to be welded), which is a widely accepted criterion. However, when the WC was too low, for example, a WC of 6 kA, the nugget formed was too small to achieve effective joining. The increase rate of the nugget was small in the range of larger WCs, whereas it increased faster in the range of smaller WCs. This is because the larger the nugget was, the nugget tip was closer to the upper A6061 sheet. Due to the large thermal conductivity of the Al alloy, more heat was lost through A6061 during welding, which resulted in a smaller growth rate of the nugget.

Secondly, the interface between the rivet shank and the upper A6061 sheet lost its original vertical characteristics and showed an arc shape. This is a result of electrode pressure during welding. The rivet shank in a high-temperature state underwent plastic deformation and was upset under the electrode pressure during welding. The degree of plastic deformation of the rivet shank was related to the WC used. Figure 2b shows the relationship between the diameter of the rivet shank in the joint and the WC. When the WC was greater than 10 kA, the rivet shank diameter detected in the joint increased with the increase in the WC. The rivet shank hardly thickened and basically maintained its original size when the WC was less than 8 kA. The larger the WC, the larger the high-temperature plasticity zone of the rivet shank. This made the rivet shank, in the joint welded under a larger WC, thicker. The plastic deformation of the rivet shank made it tightly bond to the upper A6061 sheet, as shown in Figure 2a, which would be beneficial for improving the performance of the REW A6061/Q235 joint. Moreover, the difference between rivet shank diameter and nugget diameter decreased with the increase in the WC, as shown in Figure 2b. This not only slowed down the nugget growth as previously mentioned but also affected the interfacial microstructure between the rivet shank and the upper A6061 sheet, as described below.

Thirdly, indentations were observed on the rivet tip and lower sheet side of the joint. This is also the result of the plastic deformation of the metal in the welding zone under the electrode pressure. Fourthly, a hole was observed in the central region of the nugget, and its cross-section was elliptical. According to the size and location of the hole, it was considered to be a thermal shrinkage hole. When the WC was cut off, the molten metal first began to solidify from its periphery. Due to the shrinkage strain in the solidification process, there was insufficient metal in the nugget cavity; thus, a hole formed in the nugget center, which was the final solidification zone [25]. During the holding phase of welding, the welding zone was pressed under electrode pressure so that the holes formed were flattened. In addition, splashing during welding can also reduce the metal in the nugget cavity, which can cause serious holes to form in the nugget. When the thickness of the welded plate is too large, the material cannot easily undergo plastic deformation under the electrode pressure, which results in insufficient contact between the rivet shank and the lower plate. Severe splashes occur when the WC is switched on. Therefore, this method is not suitable for welding thick plates.

In order to further explore the microstructure of the REW A6061/Q235 joint, the boundary region of the nugget (A location in Figure 2a), the inner region of the nugget (B location in Figure 2a), and the interfacial region between the rivet shank and the upper A6061 sheet (C location in Figure 2a) were enlarged and observed. The details are described below.

Figure 2c shows a simulated cross-section of the REW A6061/Q235 joint at the moment when the WC was cut off. In the calculation, the selected welding conditions were a WC of 14 kA, a WT of 300 ms, and an electrode pressure of 3.0 kN. By comparing the simulation results with the cross-section of the joint, as shown in Figure 2a, it can be found that they have good consistency in terms of the nugget diameter and thickness, but there were some slight differences in the plastic deformation of the joint. For example, the nugget tip bulged outward on the cross-section of the joint due to plastic deformation, while the simulation results show that the nugget tip was concave. In addition, electrode indentations and holes were not present in the simulation results. Although this does not affect the overall analysis, there needs to be further optimized modeling in the future, and the influence of plastic deformation should be strengthened.

**Figure 3:**
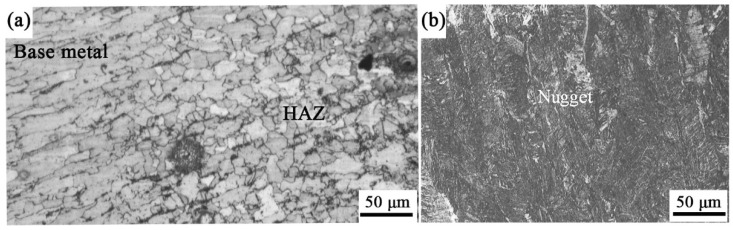
Metallographic photographs (**a**,**b**) taken from locations A and B in Figure 2, respectively.

Figure 3a,b show photographs taken from locations A and B in Figure 2, respectively. As displayed in Figure 3a, the microstructure of the base metal (Q235 steel) shows the microstructure characteristics of the rolled sheet and the grains elongated along the roll direction. The nugget was mainly composed of columnar crystals, as displayed in Figure 3b, which is a typical microstructure. The grains in the nugget were coarser than those in the base metal. The direction of these coarse columnar crystals was about parallel to the axial direction of the rivet shank. During the heating process of welding, the zone near the interface between the rivet shank and the lower sheet Q235 melted. The molten metal cooled and crystallized when the WC was cut off. The direction of grain growth was opposite to the direction of heat dissipation that was mainly carried out along the two water-cooled electrodes during REW.

Moreover, it can be seen from Figure 3a that the grains of the heat-affected zone (HAZ) were equiaxed. The grain size was in the range of 5 μm~30 μm, which was not uniform in the HAZ. During heating, part of the base metal underwent phase transformation and recrystallization, which formed austenite with fine grains. After cooling, these austenitic grains were transformed into fine pearlite and ferrite. Meanwhile, the non-austenitizing ferrite was also heated to grow and form a large equiaxed grain. However, they were finer than the columnar crystals in the nugget because no melting occurred in the HAZ. These reveal that a nugget of homogeneous metal was formed between the rivet shank and lower sheet, which made a reliable connection between them able to be achieved.

**Figure 4:**
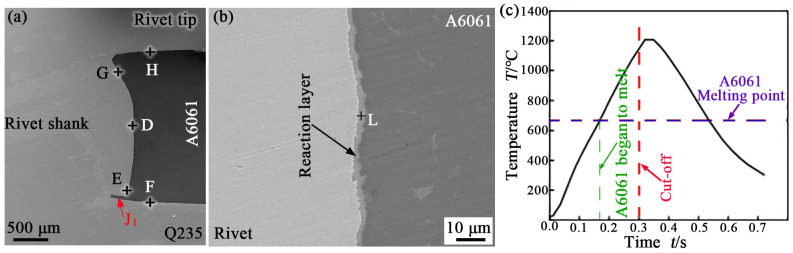
Larger view of the C region in Figure 2 (**a**); SEM image at location of D in Figure 4 (**b**); calculated temperature history curve (**c**).

Figure 4a displays a larger view of the C region in Figure 2a. As shown, the joining between the upper A6061 sheet and the rivet and between the upper A6061 sheet and the lower sheet Q235 were relatively tight. Some characteristic regions at the interface of the dissimilar materials in the joint were observed using SEM. They are the D, E, and G locations at the interface between the upper sheet and the rivet shank, the H location at the interface between the rivet tip and the upper sheet, and the F location at the interface between the upper sheet and the lower sheet, as shown in Figure 4a. Figure 4b displays an SEM image taken from the location of D in Figure 4a. At the interface, a reaction layer of about 1.6 μm in thickness was observed. The composition analysis results of the reaction layer at the L location in Figure 4b are listed in Table 2. The results show that the reaction layer was mainly composed of IMC Fe_2_Al_5_. Moreover, a gap was observed at the outer edge of the rivet shank marked by J_1_, as shown in Figure 4a, and was about 250 μm wide. The formation of the gap was mainly due to the uneven grinding of the end face of the rivet shank during rivet processing.

Figure 4c shows the calculated temperature history curve at the D location in Figure 4a. It displays that the peak temperature here reached 1200 °C. This reveals that the rivet shank near the interface did not melt during the welding process, but A6061 near the interface melted. As shown in Figure 4c, A6061 near the interface began to melt after 168 ms of heating. The duration of temperature above the melting point of A6061 (hereafter called high-temperature duration) was approximately 353 ms at the position. These indicate that a metallurgical reaction occurred between the solid steel and the liquid Al alloy at the D position of the interface during welding. Moreover, it may be noted from Figure 4c that the temperature here would continue to rise after the WC was cut off. There are two main reasons for this. Firstly, there was a lag time for heat conduction. This is because there is a distance between the position and the heat production center that is considered to be the center of the interface between the end of the rivet shank and the lower sheet. Secondly, molten metal released latent heat when it solidified. The latent heat also heated the metal in the zone around the nugget.

**Figure 5:**
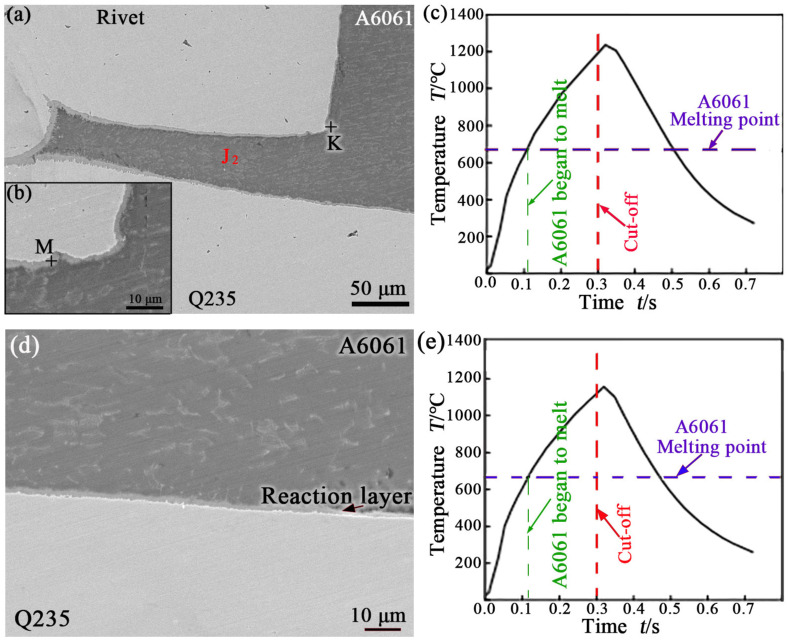
SEM image taken from the location of E in Figure 4 (**a**); larger view of the K region in Figure 5 (**b**); calculated temperature history curve at E (**c**); SEM image taken from the location of F in Figure 4 (**d**); calculated temperature history curve at F (**e**).

Figure 5a displays an SEM image taken from the location of E in Figure 4a. It should be noted that the gap (J_2_ location) between the end of the rivet shank and the lower sheet was filled with aluminum alloy. A reaction layer was also observed at the periphery of the gap, as shown in Figure 5a. The upper sheet of A6061 outside the rivet shank was heated, expanded, and squeezed into the gap during welding, and a metallurgical reaction between it and the steel (the rivet; the lower sheet of Q235) around it occurred to form reactants. This could prevent the edge notch effect and avoid the occurrence of stress concentration at the gap. Figure 5b,c show a larger view of the K region in Figure 5a and a temperature history curve at the E position in Figure 4a during welding, respectively. At the vertical interface (between the rivet shank and A6061), a reaction layer of about 2.0 μm in thickness was observed. The composition analysis results of the reaction layer at the M location in Figure 5b are also listed in Table 2. The results show that the reaction layer was mainly composed of IMC Fe_2_Al_5_. The calculated temperature history curve shows that the peak temperature here reached 1237 °C. As shown in Figure 5c, the A6061 near the interface began to melt after 110 ms of heating. The high-temperature duration was approximately 392 ms at this position.

Figure 5d displays an SEM image taken from the location of F in Figure 4a. At the interface between the upper sheet and the lower sheet, a reaction layer of about 1.4 μm in thickness was observed. The calculated temperature history curve (Figure 5e) shows that the peak temperature here reached 1123 °C. A6061 near the interface began to melt after 117 ms of heating. The high-temperature duration was approximately 354 ms at this position, as shown in Figure 5e.

By comparing Figure 5c,e, it can be found that although interfacial metallurgical reactions occurred between the solid steel and the liquid Al alloy at E and F positions (Figure 4a) during REW, their thermal history curves were slightly different. At the E position, the peak temperature was higher, the high-temperature duration was longer, and A6061 near the interface began to melt earlier compared with the F position. This is because the distance from the F position to the center of the rivet axis is farther than that from the E position. These thermal historical differences are also the reason why the reaction layer formed at the E position was thicker than that at the F position, as shown in Figure 5.

**Figure 6:**
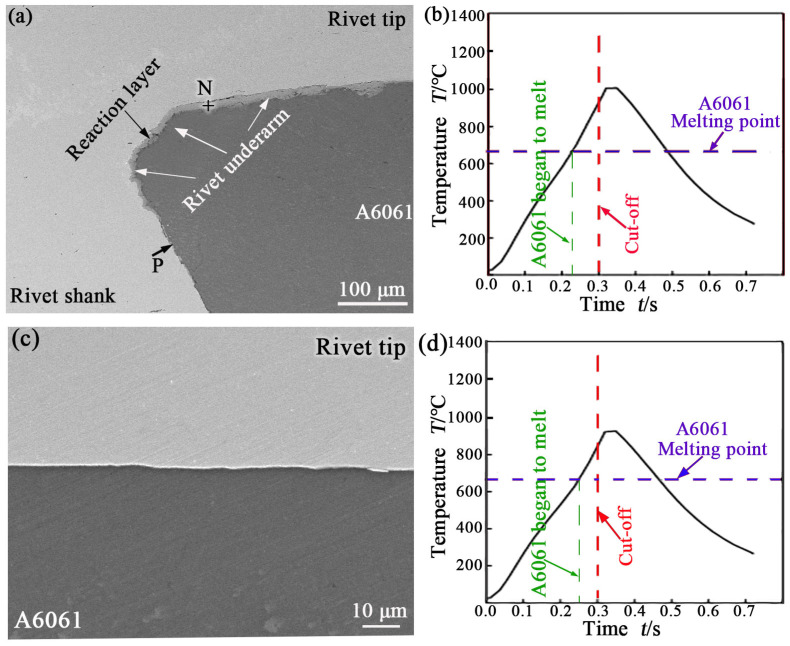
SEM image at location of G in Figure 4 (**a**); calculated temperature history curve at G (**b**); SEM image at location of H in Figure 4 (**c**); calculated temperature history curve at H (**d**).

Figure 6a displays an SEM image taken from the location of G in Figure 4a. The calculated temperature history curve (Figure 6b) shows that the peak temperature here reached 1000 °C, A6061 near the interface began to melt after 228 ms of heating, and the high-temperature duration was approximately 260 ms at this position. The interface between the rivet shank and the upper A6061 sheet in Figure 6a is vertical. The top part of the interface is slightly different from the lower part. The reaction layer was observed at the upper part of the interface (above position P in Figure 6a), but not at the lower part of the interface. The composition analysis results of the reaction layer at the N location in Figure 6a are also listed in Table 2. The results show that the reaction layer was mainly composed of IMC Fe_2_Al_5_.

Figure 6c displays an SEM image taken from the location of H in Figure 4a. The rivet tip was closely bonded to the upper A6061 sheet, but no reactants were generated at this interface. The calculated temperature history curve (Figure 6d) shows that the peak temperature here reached 890 °C, A6061 near the interface began to melt after 252 ms of heating, and the high-temperature duration was approximately 224 ms at this position. The lower peak temperature and shorter high-temperature duration at G and H positions in Figure 4a are considered to be the reasons why no reactants were observed at the two positions.

In addition, a reaction layer of about 12 μm in thickness was observed at the rivet underarm, as shown in Figure 6a. There are three main reasons for the thicker reaction layer formed at the interface. First, in terms of spatial position, the distance from the rivet underarm to the rivet axis was closer than that from the D position (Figure 4a) due to deformation, as shown in Figure 4a. This makes it easier for the rivet underarm to receive heat by conduction. Second, the rivet underarm is not easy to dissipate heat. This is because it is topped with a rivet tip (steel), which has poor thermal conductivity than aluminum alloy. Therefore, the peak temperature was higher, and the high-temperature duration was longer at the rivet underarm, which made the reaction layer formed in the region thicker. Unfortunately, the temperature history curve in this region could not be accurately extracted due to the influence of deformation, which needs to be further studied in the future. Third, the reactants started to form early here. The reactants were formed as a result of the mutual diffusion of atoms on both sides of the interface, which required close contact between the metals on both sides of the interface. Since the interface between the rivet shank and the upper A6061 sheet in Figure 6a is vertical, only the thermal expansion or upsetting of the rivet shank can promote the close contact of the vertical interface in the joint during REW. Therefore, there was a lag period for atomic diffusion through this vertical interface. On the other hand, in the pre-pressing stage, when only electrode pressure was applied without the WC, the interface under the rivet underarm was in close contact. This provided a guarantee for atomic diffusion through the interface.

Orderly at G, D, and E positions in Figure 4a, it can be found that the peak temperature and the high-temperature duration increased, and the melting time of A6061 near the interface advanced by comparing Figure 4c, Figure 5c, and Figure 6b. The WC flowed from the upper electrode, along the rivet (the rivet tip and shank) to the lower sheet, and then to the lower electrode during REW because there were fewer contact interfaces and smaller contact resistance in the WC path in this case. Therefore, Joule heat was mainly generated at the interface between the rivet shank and the lower sheet in the early stage of welding because there was contact resistance. Thus, the metal around the interface between the rivet shank and the lower sheet was heated. Although the contact resistance between the rivet shank and the lower sheet disappeared soon after electrization heating, it induced an increase in the temperature and resistivity of the metal surrounding it. This caused the area of heating and melting to expand as the electrification continued. Because the distance from the interface between the rivet shank and lower sheet to the lower electrode (2 mm) was shorter than that to the upper electrode (4 mm), the melting area mainly expanded to the side of the rivet tip (upper electrode) during REW under the cooling action of electrodes. This not only caused the nugget to skew to one side of the rivet tip (as described earlier) but also resulted in different thermal histories at various positions in the interface between the rivet shank and the upper sheet. As a result, the thickness of the reaction layer generated at the interface between the rivet shank and the upper sheet (except the zone of the rivet underarm) varied with its position, as shown in Figure 4b, Figure 5b, and Figure 6a. When the REW method is used to weld thicker plates (Al alloy and steel), the distribution of the reaction layer thickness along the interface between the rivet shank and the upper sheet is similar, but the formed nugget is more inclined to the side of the rivet tip.

As previously mentioned, the reaction layer generated at the interface between aluminum alloy and steel in the joint was mainly composed of IMC Fe_2_Al_5_. These results are consistent with previous reports [26], and the reasons are detailed in reference [27].

**Figure 7:**
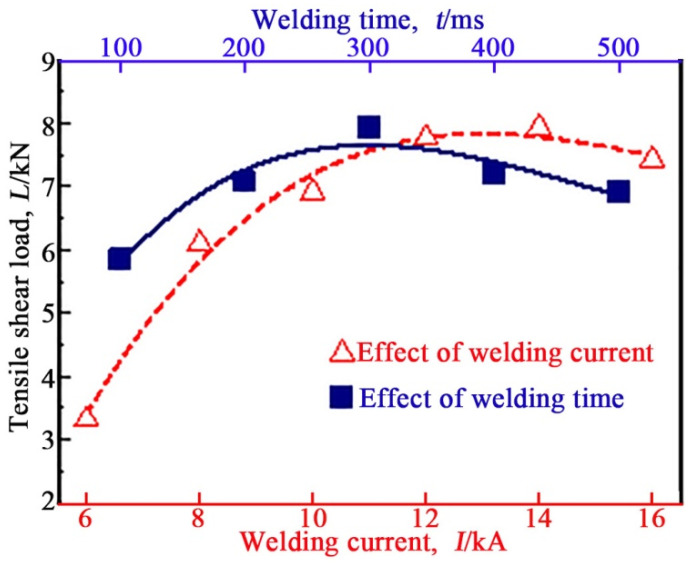
Effects of WC and WT on the tensile shear load of REW A6061/Q235 joint.

Figure 7 displays the effects of WC and WT on the tensile shear load of the REW A6061/Q235 joint. The tensile shear load of the REW A6061/Q235 joint first increased and then slowly decreased with the increase in WC. When the welding current was 14 kA, the tensile shear load of the REW A6061/Q235 joint reached a maximum of 7.93 kN. In the WC range of 6 kA~14 kA, the nugget diameter of the REW A6061/Q235 joint increased with the increase in WC, as displayed in Figure 2c. This is the reason why the tensile shear load of the REW A6061/Q235 joint increased with increases in WC. When the WC was greater than 14 kA, the tensile shear load of the REW A6061/Q235 joint decreased slightly with the increase in WC, despite the increase in the nugget diameter. This is mainly because excessive WC can cause the reaction layer at the interface between Al alloy and steel in the joint to become thicker. As displayed in Figure 7, the tensile shear load of the REW A6061/Q235 joint also first increased and then decreased with the increase in WT. The reason was similar to the above effect of WC.

**Figure 8:**
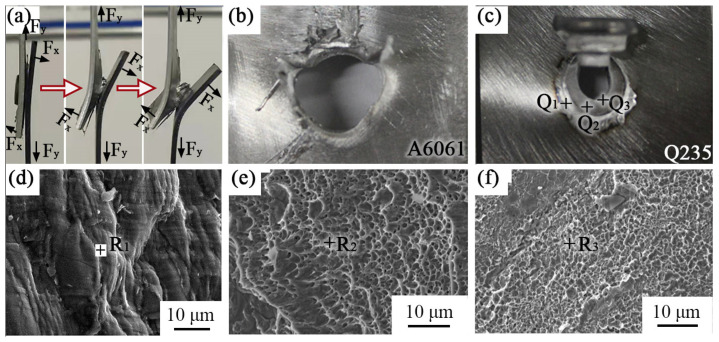
Photographs of failure process and fracture images of the joint. (**a**) Failure process photographs; (**b**) fracture of A6061 side; (**c**) fracture of Q235 side; (**d**–**f**) taken from Q_1_, Q_2_, and Q_3_ in (**c**), respectively.

The fracture modes of the REW A6061/Q235 joint mainly included interface tearing and button fracture. Interface tearing means that the joint was damaged from the interface between the rivet shank and the lower sheet. This was mainly due to the small nugget formed between the rivet shank and the lower sheet. When the WC was lower than 8 kA, the fracture of the REW A6061/Q235 joint was mainly interface tearing. When the WC was larger than 8 kA, the failure mode of the REW A6061/Q235 joint was button failure. Figure 8 shows failure process photographs and fracture images of the REW A6061/Q235 joint. During the process of tensile shear testing, the joint not only withstood a shear force of Fy but also withstood a force of Fx, as displayed in Figure 8a. In this case, Figure 8b,c show the fracture on the A6061 and Q235 sides of the joint, respectively. There were roughly three stages for button failure of the joint. First, the joint was torn from the interface of the upper A6061 sheet and the lower Q235 sheet under the action of load. Then, the rivet shank with the nugget was pulled out from the lower Q235 sheet as the direction of the Fx force changed. Finally, the pre-machined hole became larger in the upper A6061 sheet due to the plastic deformation of the metal around it, which caused the rivet tip to pull out from the upper A6061 sheet. Figure 8d–f show the SEM image taken from Q_1_, Q_2_, and Q_3_ in Figure 8c, respectively. Although the composition analysis at R_1_, R_2_, and R_3_ in Figure 8 shows that these regions were Al alloy, their morphology was quite different. Figure 8d shows that the fracture in this region was relatively flat, while more dimples were observed in Figure 8e,f. From the fracture morphology, composition analysis results, and the position shown in Figure 8c, it can be inferred that the fracture at Q_1_ occurred from the aluminum alloy splash squeezed between the upper sheet and the lower sheet (outside the rivet in the joint), and the fracture at Q_2_ and Q_3_ occurred within the upper A6061 sheet. When the WC used was too large or the WT was too long, the reaction layer at the interface between the upper sheet and the lower sheet was thicker, which might cause the failure crack to occur at the reaction layer between the upper sheet and the lower sheet outside the rivet shank in the joint. It is because of this that the tensile shear load of the REW A6061/Q235 joint decreased with increases in the WC and WT when the WC was too large or the WT was too long.

## 4. Conclusions

Through this study, the following conclusions can be drawn:

1. A nugget was formed between the rivet shank and the lower sheet when A6061 Al alloy and Q235 steel were welded by REW with a steel rivet.
2. The smaller the distance from the position to the lower sheet along the interface between the rivet shank and upper sheet, the longer the high-temperature duration and the higher the peak temperature during welding. At the junction of the rivet shank, upper sheet, and lower sheet in the joint, the high-temperature duration was the longest, at about 392 ms, and the peak temperature was the highest, at about 1237 °C.
3. The smaller the distance from the position to the lower sheet along the interface between the rivet shank and the upper sheet in the joint, the thicker the reaction layer generated there. The thickness of the reaction layer was about 2.0 μm at the junction of the rivet shank, upper sheet, and lower sheet in the joint.
4. With increases in WC and WT, the tensile shear load of the joint increased first and then decreased slightly. When the WC was 14 kA and the WT was 300 ms, the tensile shear load of the joint reached a maximum of 7.93 kN.

## Figures and Tables

**Table 1:** Welding parameters used.

Series Title	WC (kA)	WT (ms)	Electrode Pressure (kN)	Electrode Tip Diameter (mm)
Serie I	6~16	300	3.0	6
Serie II	14	100~500	3.0	6

**Table 2:** EDS results.

Position	Composition (at.%)	
	Fe	Al
L	26.25	73.25
M	25.99	74.01
N	27.84	72.16

## Data Availability

The data are contained within the article.
